# Supplementary figures and images for: Asymmetric localization of the cell division machinery during Bacillus subtilis sporulation
Source: eLife. 2021 May 21;10:e62204. doi: 10.7554/eLife.62204 (PMC8192124; doi:10.7554/eLife.62204)

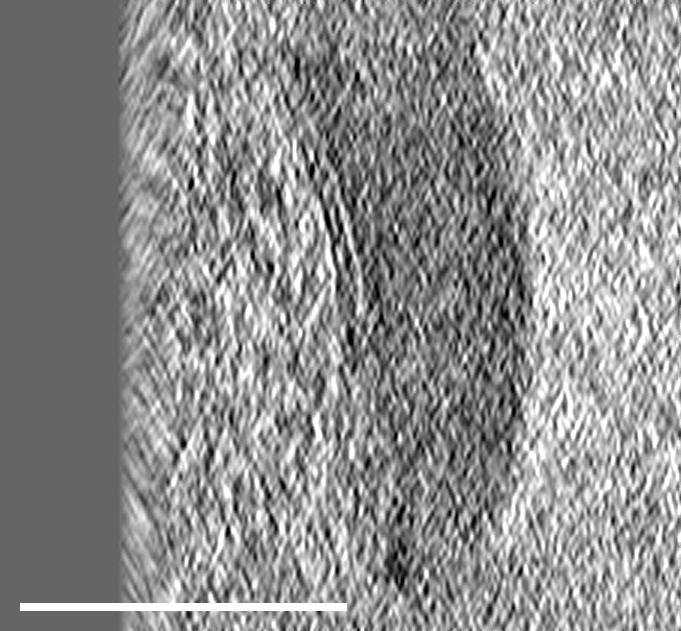

Supplement: Source code 1. [file elife-62204-code1.zip › example_input/20161115_L4T2_oneside_200.tif]

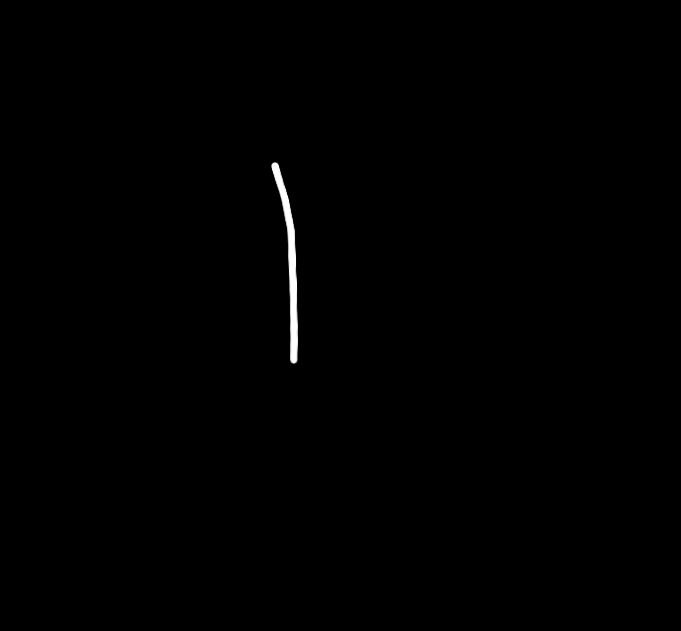

Supplement: Source code 1. [file elife-62204-code1.zip › example_input/20161115_L4T2_oneside_200_cytoplasm.tif]

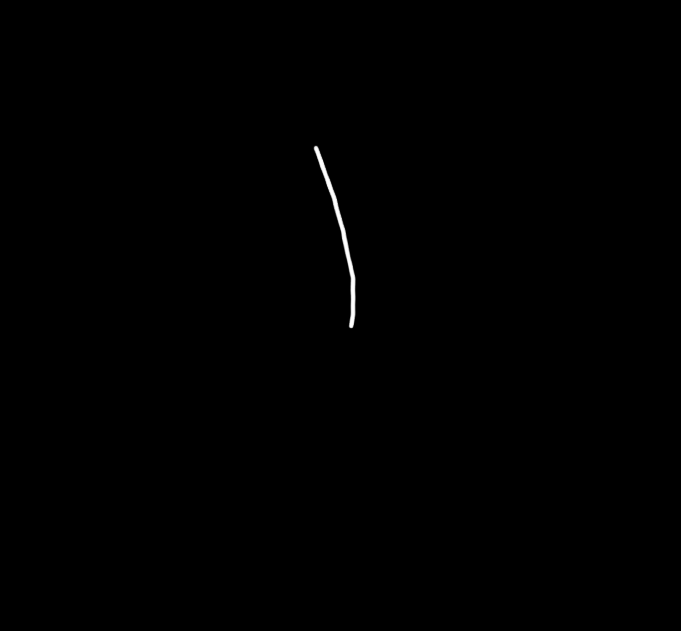

Supplement: Source code 1. [file elife-62204-code1.zip › example_input/20161115_L4T2_oneside_200_ftsA.tif]

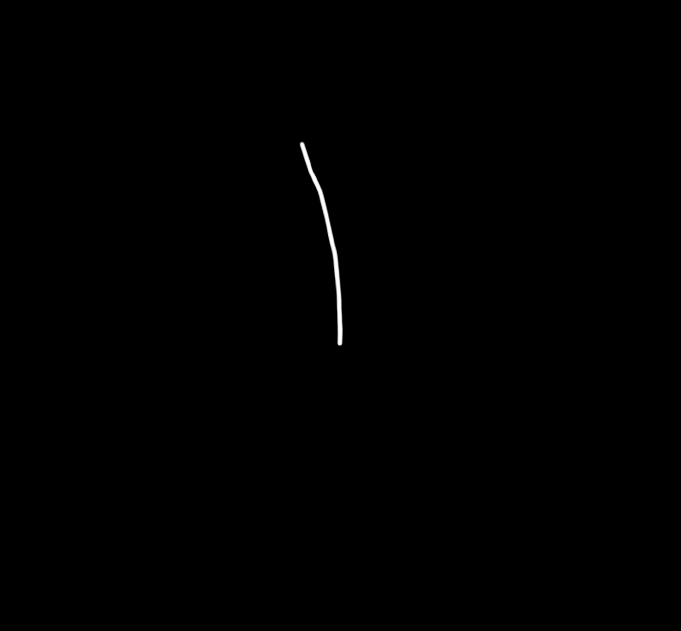

Supplement: Source code 1. [file elife-62204-code1.zip › example_input/20161115_L4T2_oneside_200_ftsZ.tif]

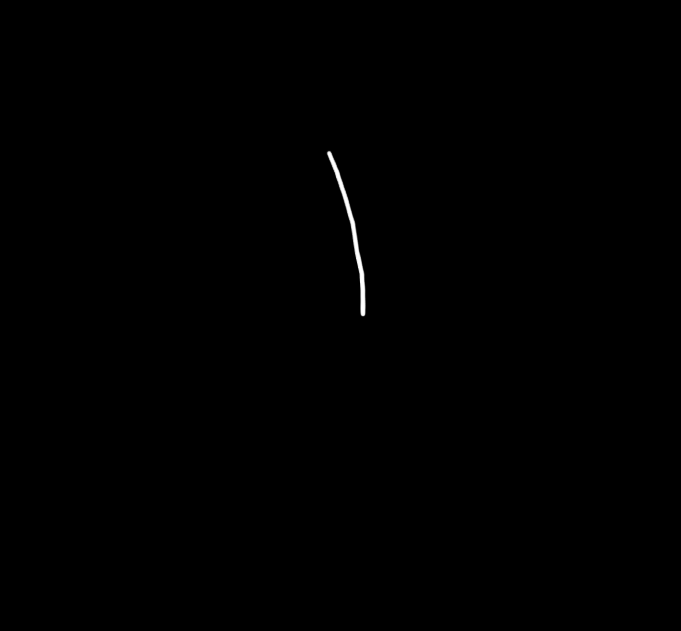

Supplement: Source code 1. [file elife-62204-code1.zip › example_input/20161115_L4T2_oneside_200_membrane.tif]

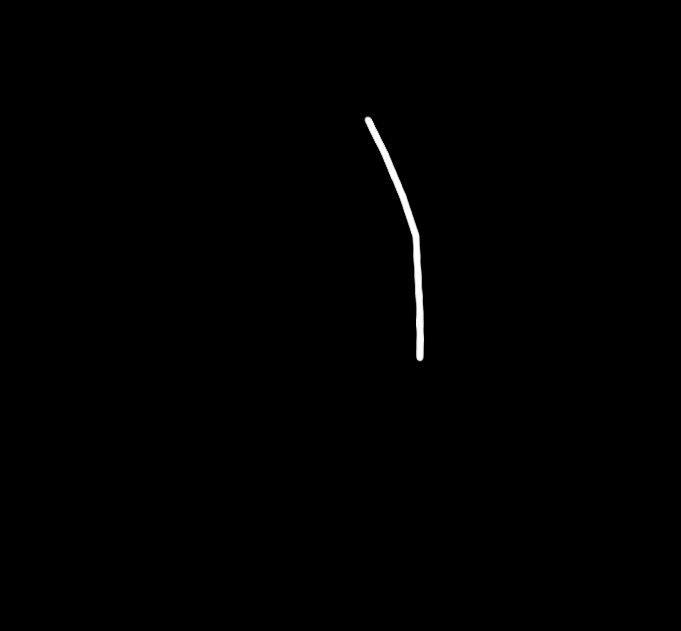

Supplement: Source code 1. [file elife-62204-code1.zip › example_input/20161115_L4T2_oneside_200_PG.tif]

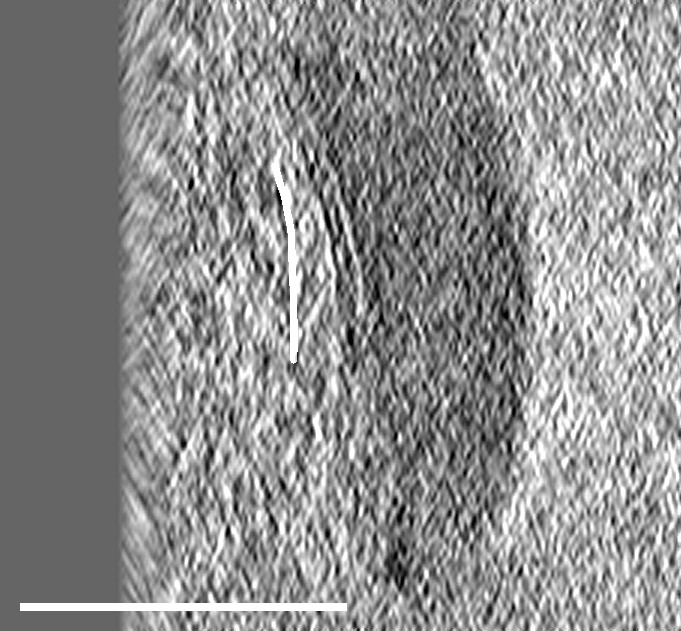

Supplement: Source code 1. [file elife-62204-code1.zip › example_output/20161115_L4T2_oneside_200_cytoplasm.tif]

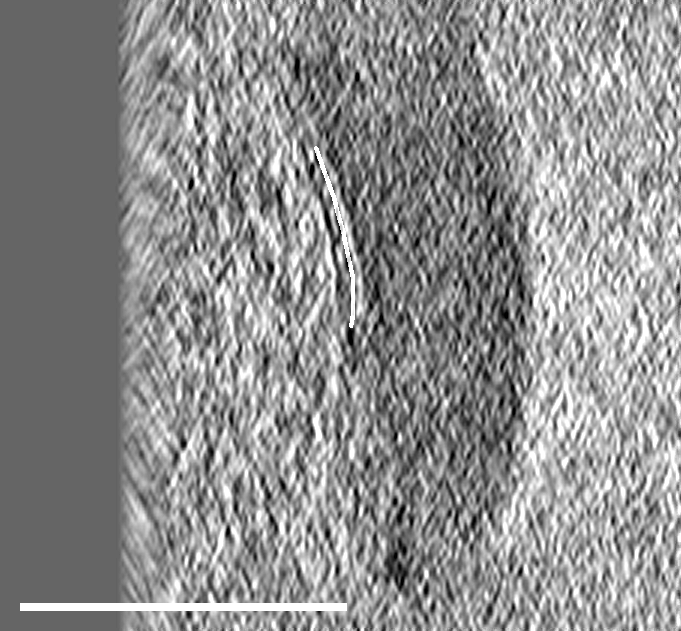

Supplement: Source code 1. [file elife-62204-code1.zip › example_output/20161115_L4T2_oneside_200_ftsA.tif]

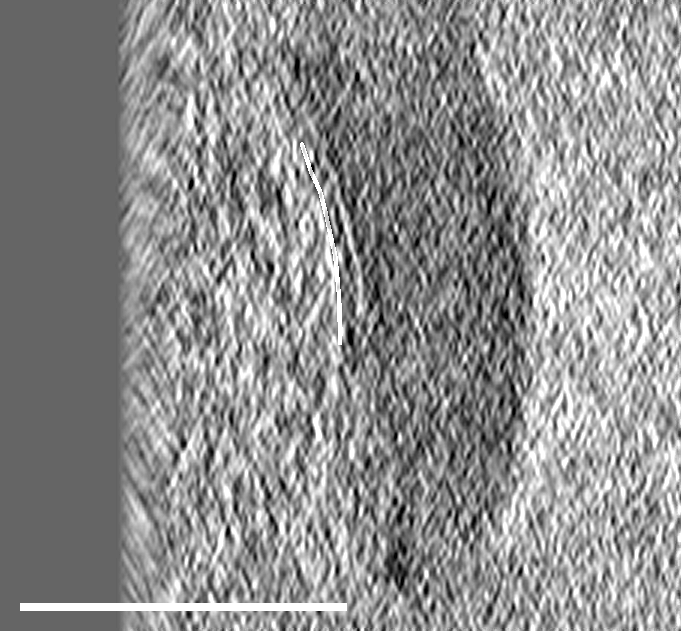

Supplement: Source code 1. [file elife-62204-code1.zip › example_output/20161115_L4T2_oneside_200_ftsZ.tif]

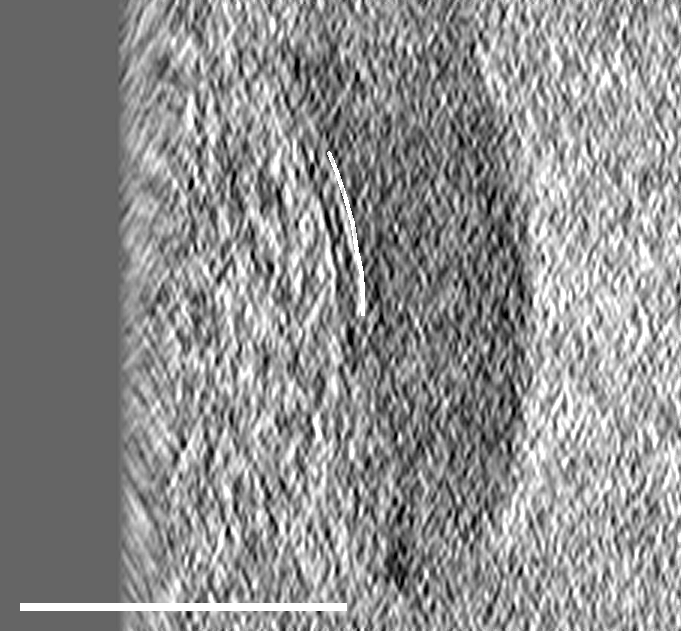

Supplement: Source code 1. [file elife-62204-code1.zip › example_output/20161115_L4T2_oneside_200_membrane.tif]

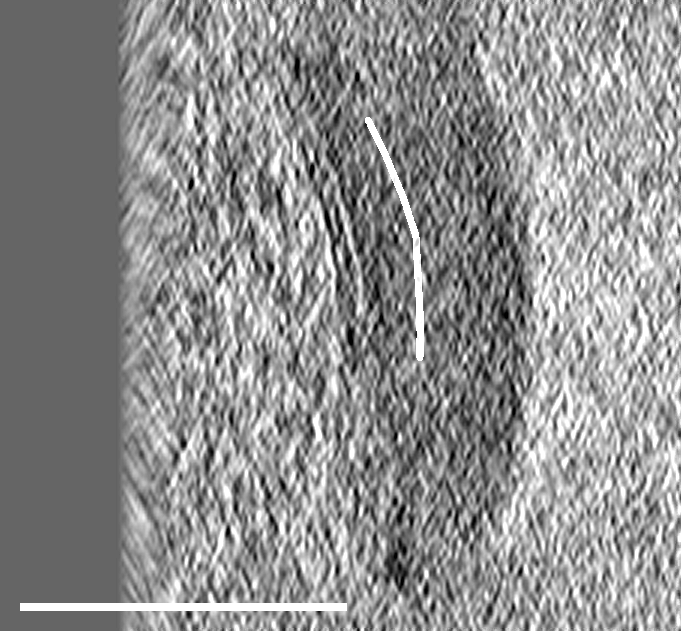

Supplement: Source code 1. [file elife-62204-code1.zip › example_output/20161115_L4T2_oneside_200_PG.tif]

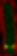

Supplement: Source code 2. [file elife-62204-code2.zip › KK421_masked/KK421_1.tif]

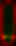

Supplement: Source code 2. [file elife-62204-code2.zip › KK421_masked/KK421_10.tif]

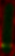

Supplement: Source code 2. [file elife-62204-code2.zip › KK421_masked/KK421_11.tif]

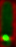

Supplement: Source code 2. [file elife-62204-code2.zip › KK421_masked/KK421_12.tif]

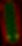

Supplement: Source code 2. [file elife-62204-code2.zip › KK421_masked/KK421_13.tif]

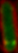

Supplement: Source code 2. [file elife-62204-code2.zip › KK421_masked/KK421_14.tif]

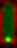

Supplement: Source code 2. [file elife-62204-code2.zip › KK421_masked/KK421_15.tif]

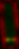

Supplement: Source code 2. [file elife-62204-code2.zip › KK421_masked/KK421_2.tif]

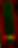

Supplement: Source code 2. [file elife-62204-code2.zip › KK421_masked/KK421_3.tif]

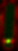

Supplement: Source code 2. [file elife-62204-code2.zip › KK421_masked/KK421_4.tif]

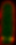

Supplement: Source code 2. [file elife-62204-code2.zip › KK421_masked/KK421_5.tif]

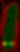

Supplement: Source code 2. [file elife-62204-code2.zip › KK421_masked/KK421_6.tif]

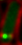

Supplement: Source code 2. [file elife-62204-code2.zip › KK421_masked/KK421_7.tif]

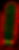

Supplement: Source code 2. [file elife-62204-code2.zip › KK421_masked/KK421_8.tif]

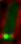

Supplement: Source code 2. [file elife-62204-code2.zip › KK421_masked/KK421_9.tif]
